# Supplementary material for: Detailed molecular and epigenetic characterization of the pig IPEC-J2 and chicken SL-29 cell lines
Source: iScience. 2023 Feb 20;26(3):106252. doi: 10.1016/j.isci.2023.106252 (PMC10018572; doi:10.1016/j.isci.2023.106252)
Supplement: Data S1. Complete homer output for identified motifs in Pig IPECJ-2, related to Table 2 — Homer motif analysis results for histone modifications H3K4me1, H3K4me3, H3K27ac, and enhancer elements of pig IPECJ2 cell line. P-values >1e-10 are possible false positives. Within each folder (e.g. peak_files_CTCF) are the html files showing the identified motifs when using homer (e.g. homerResults.html). [file mmc2.zip › S5/Pig_IPECJ_2/peak_files_H3K27ac/homerResults.html]

peak\_files\_H3K27ac/ - Homer de novo Motif Results


# Homer *de novo* Motif Results (peak\_files\_H3K27ac/)

Known Motif Enrichment Results  
Gene Ontology Enrichment Results  
If Homer is having trouble matching a motif to a known motif, try copy/pasting the matrix file into
STAMP  
More information on motif finding results: HOMER
| Description of Results
| Tips
  
Total target sequences = 52582  
Total background sequences = 51862  
\* - possible false positive  

|  |  |  |  |  |  |  |  |  |
| --- | --- | --- | --- | --- | --- | --- | --- | --- |
| Rank | Motif | P-value | log P-pvalue | % of Targets | % of Background | STD(Bg STD) | Best Match/Details | Motif File |
| 1 | T G C A G A C T A C G T A C G T A G C T G T A C T A G C G A T C A T G C A T C G | 1e-141 | -3.261e+02 | 49.69% | 44.17% | 84.6bp (91.0bp) | E2F(E2F)/Hela-CellCycle-Expression/Homer(0.752) More Information \| Similar Motifs Found | motif file (matrix) |
| 2 | A C T G A G C T C G A T T G C A T A C G A T C G T G A C C T G A | 1e-120 | -2.768e+02 | 50.64% | 45.56% | 85.0bp (89.3bp) | ZNF449/MA1656.1/Jaspar(0.690) More Information \| Similar Motifs Found | motif file (matrix) |
| 3 | C G T A T C G A G C T A T A C G A C T G A T C G A G T C A C G T | 1e-111 | -2.558e+02 | 46.14% | 41.29% | 84.6bp (90.4bp) | REL/MA0101.1/Jaspar(0.572) More Information \| Similar Motifs Found | motif file (matrix) |
| 4 | A G T C G T A C A C T G A T C G C G A T C G T A T G C A C A T G | 1e-110 | -2.543e+02 | 23.54% | 19.57% | 84.7bp (91.4bp) | SD0001.1\_at\_AC\_acceptor/Jaspar(0.816) More Information \| Similar Motifs Found | motif file (matrix) |
| 5 | C G T A A G C T A T C G A T C G A G T C A T C G A T C G G T A C A T C G A T C G | 1e-104 | -2.414e+02 | 4.41% | 2.73% | 89.7bp (95.6bp) | YY2/MA0748.2/Jaspar(0.908) More Information \| Similar Motifs Found | motif file (matrix) |
| 6 | T C G A T A G C G A T C T A C G C A T G C T A G G C A T A G C T A G C T A C G T | 1e-103 | -2.392e+02 | 36.69% | 32.22% | 84.5bp (89.7bp) | Prdm15/MA1616.1/Jaspar(0.793) More Information \| Similar Motifs Found | motif file (matrix) |
| 7 | C G A T G C A T T G C A A T G C A G C T A G T C C T A G G T A C | 1e-93 | -2.160e+02 | 29.63% | 25.64% | 85.9bp (91.7bp) | E2F7(E2F)/Hela-E2F7-ChIP-Seq(GSE32673)/Homer(0.622) More Information \| Similar Motifs Found | motif file (matrix) |
| 8 | G T A C C G T A A T G C C T A G G C A T A C G T G A C T A G T C | 1e-84 | -1.953e+02 | 49.63% | 45.37% | 86.1bp (89.4bp) | TEAD2/MA1121.1/Jaspar(0.636) More Information \| Similar Motifs Found | motif file (matrix) |
| 9 | T G A C T C A G T A C G A G C T A G C T C G A T A T C G C G T A T C G A T C G A | 1e-84 | -1.951e+02 | 53.90% | 49.64% | 85.1bp (90.7bp) | CHR(?)/Hela-CellCycle-Expression/Homer(0.834) More Information \| Similar Motifs Found | motif file (matrix) |
| 10 | C T G A C T A G A C T G A G T C G C T A T C A G G C T A C G A T G A T C G C A T A C G T A G C T A G C T | 1e-83 | -1.929e+02 | 0.12% | 0.00% | 80.6bp (81.0bp) | PB0116.1\_Elf3\_2/Jaspar(0.601) More Information \| Similar Motifs Found | motif file (matrix) |
| 11 | T G C A A T C G G T A C G C A T C T G A T C A G G A T C C G A T | 1e-81 | -1.872e+02 | 43.40% | 39.30% | 85.1bp (89.8bp) | PB0154.1\_Osr1\_2/Jaspar(0.636) More Information \| Similar Motifs Found | motif file (matrix) |
| 12 | A C T G C G A T G A C T A G T C C T A G C A T G C T G A T C A G | 1e-73 | -1.683e+02 | 39.54% | 35.72% | 84.4bp (91.3bp) | PB0115.1\_Ehf\_2/Jaspar(0.697) More Information \| Similar Motifs Found | motif file (matrix) |
| 13 | T C G A T C A G C A G T A G C T G A C T G A T C C T G A T A G C G A T C A C T G | 1e-72 | -1.669e+02 | 23.04% | 19.84% | 84.1bp (91.1bp) | IRF4(IRF)/GM12878-IRF4-ChIP-Seq(GSE32465)/Homer(0.680) More Information \| Similar Motifs Found | motif file (matrix) |
| 14 | A C T G G T A C A C T G T A G C C A T G C G T A A G T C T A G C | 1e-55 | -1.288e+02 | 18.79% | 16.20% | 85.2bp (94.7bp) | ZBED1/MA0749.1/Jaspar(0.674) More Information \| Similar Motifs Found | motif file (matrix) |
| 15 | A T G C A T G C T C A G C A T G A C G T A G C T A G T C T A G C A T C G T A C G | 1e-50 | -1.156e+02 | 20.20% | 17.67% | 84.6bp (95.1bp) | Elk4(ETS)/Hela-Elk4-ChIP-Seq(GSE31477)/Homer(0.775) More Information \| Similar Motifs Found | motif file (matrix) |
| 16 | A G T C C A T G G C A T C T G A C T A G C T A G T C A G T C G A C T G A C G T A | 1e-49 | -1.146e+02 | 10.45% | 8.58% | 84.6bp (92.6bp) | NFATC3/MA0625.1/Jaspar(0.664) More Information \| Similar Motifs Found | motif file (matrix) |
| 17 | G C A T G C T A T A C G A C G T A G C T G A T C T A C G T C A G T A C G T A G C | 1e-49 | -1.144e+02 | 11.01% | 9.09% | 83.7bp (92.2bp) | THAP1/MA0597.1/Jaspar(0.628) More Information \| Similar Motifs Found | motif file (matrix) |
| 18 | A C G T A C G T C A G T C T G A A T C G C T G A C A T G T A G C C A T G G T A C | 1e-46 | -1.075e+02 | 16.98% | 14.71% | 84.0bp (92.4bp) | PH0158.1\_Rhox11\_2/Jaspar(0.662) More Information \| Similar Motifs Found | motif file (matrix) |
| 19 | A C G T A G C T A C G T T G C A T A G C G T A C C G T A A C T G A G C T C A G T C A T G C T A G C A T G | 1e-45 | -1.044e+02 | 0.10% | 0.01% | 77.3bp (38.8bp) | AMYB(HTH)/Testes-AMYB-ChIP-Seq(GSE44588)/Homer(0.651) More Information \| Similar Motifs Found | motif file (matrix) |
| 20 | A T G C A G T C C G T A T C G A A C T G C G T A A C T G A T C G G A T C A C G T A T G C A C G T A C G T | 1e-43 | -9.931e+01 | 0.09% | 0.01% | 79.4bp (36.4bp) | Zac1(Zf)/Neuro2A-Plagl1-ChIP-Seq(GSE75942)/Homer(0.599) More Information \| Similar Motifs Found | motif file (matrix) |
| 21 | T A G C T G A C C A T G G C T A G T A C A G T C A T C G T C G A | 1e-42 | -9.744e+01 | 8.37% | 6.81% | 84.0bp (95.1bp) | HINFP(Zf)/K562-HINFP.eGFP-ChIP-Seq(Encode)/Homer(0.676) More Information \| Similar Motifs Found | motif file (matrix) |
| 22 | T G C A G C A T C T G A A G C T A G T C G T C A A T G C A G T C A G T C G A T C G T A C G T C A C G A T | 1e-41 | -9.620e+01 | 0.08% | 0.00% | 87.6bp (7.2bp) | PB0201.1\_Zfp281\_2/Jaspar(0.729) More Information \| Similar Motifs Found | motif file (matrix) |
| 23 | G C T A A G T C C G A T C T G A G T A C G A T C A C T G A G T C A G T C C T A G | 1e-41 | -9.561e+01 | 3.65% | 2.65% | 86.0bp (95.6bp) | ZBTB7B/MA0694.1/Jaspar(0.694) More Information \| Similar Motifs Found | motif file (matrix) |
| 24 | A G T C A C G T A C T G A G T C A C G T G T A C A G T C C G T A A C T G G A T C G T A C G T A C C G A T | 1e-40 | -9.312e+01 | 0.08% | 0.01% | 76.8bp (51.9bp) | POL011.1\_XCPE1/Jaspar(0.683) More Information \| Similar Motifs Found | motif file (matrix) |
| 25 | C G T A A C T G A C G T A C G T C G T A A C T G C G T A C G T A T G A C G T C A T C G A C G T A A C T G | 1e-33 | -7.668e+01 | 0.08% | 0.01% | 82.6bp (91.1bp) | Sox4(HMG)/proB-Sox4-ChIP-Seq(GSE50066)/Homer(0.679) More Information \| Similar Motifs Found | motif file (matrix) |
| 26 | A G C T C G A T T A C G A G C T A C G T A G C T A C T G T G A C A T G C A T C G | 1e-32 | -7.524e+01 | 1.52% | 0.97% | 85.9bp (90.4bp) | FOXN3/MA1489.1/Jaspar(0.726) More Information \| Similar Motifs Found | motif file (matrix) |
| 27 | T C G A A G C T A C T G C G T A A T G C A C G T G T A C C G T A | 1e-32 | -7.470e+01 | 7.09% | 5.83% | 94.6bp (87.8bp) | BATF/MA1634.1/Jaspar(0.967) More Information \| Similar Motifs Found | motif file (matrix) |
| 28 | C A T G C T A G A T G C G C T A G A C T A C G T G C A T G C T A G A T C G T A C C T G A G A C T C G T A | 1e-29 | -6.768e+01 | 0.08% | 0.01% | 87.3bp (23.2bp) | NFAT5/MA0606.1/Jaspar(0.674) More Information \| Similar Motifs Found | motif file (matrix) |
| 29 | C G A T T G C A C G T A C T A G C G A T C T A G C T A G C T G A C G T A G T A C G C A T C G T A C G A T | 1e-27 | -6.370e+01 | 0.06% | 0.01% | 76.0bp (65.8bp) | PU.1:IRF8(ETS:IRF)/pDC-Irf8-ChIP-Seq(GSE66899)/Homer(0.702) More Information \| Similar Motifs Found | motif file (matrix) |
| 30 | A T C G G T A C T C G A T A C G T G A C T A C G A T C G T A G C T C A G T C A G G T C A T A C G G A C T | 1e-27 | -6.310e+01 | 0.11% | 0.02% | 88.9bp (126.0bp) | NHLH1/MA0048.2/Jaspar(0.686) More Information \| Similar Motifs Found | motif file (matrix) |
| 31 | C G T A C T A G A G C T C T A G C T A G C G T A A C T G A C T G | 1e-27 | -6.275e+01 | 12.80% | 11.27% | 85.9bp (90.4bp) | MAZ/MA1522.1/Jaspar(0.682) More Information \| Similar Motifs Found | motif file (matrix) |
| 32 | G T C A G C A T G C A T G A C T C G T A C T A G C T G A T G A C G T A C C G T A T C A G C A G T T G C A | 1e-26 | -6.188e+01 | 0.05% | 0.00% | 84.1bp (71.9bp) | Bcl11a(Zf)/HSPC-BCL11A-ChIP-Seq(GSE104676)/Homer(0.593) More Information \| Similar Motifs Found | motif file (matrix) |
| 33 | T G A C C T A G A T C G C T G A T A G C G C T A A T C G G C A T A G T C A G T C G C A T A C G T A T G C | 1e-26 | -6.090e+01 | 0.06% | 0.01% | 68.5bp (60.2bp) | Znf263(Zf)/K562-Znf263-ChIP-Seq(GSE31477)/Homer(0.591) More Information \| Similar Motifs Found | motif file (matrix) |
| 34 | C G T A C G T A G T A C G T A C G A C T A G T C G A T C G A C T C A G T A C T G T C G A C A G T G T C A | 1e-24 | -5.616e+01 | 0.07% | 0.01% | 82.3bp (23.9bp) | PH0150.1\_Pou4f3/Jaspar(0.574) More Information \| Similar Motifs Found | motif file (matrix) |
| 35 | C G T A A C T G C G T A A C G T C G T A C G T A C G T A A C T G A C T G A G T C A C T G A C T G A C T G | 1e-24 | -5.541e+01 | 0.06% | 0.01% | 95.4bp (35.7bp) | GATA3(Zf)/iTreg-Gata3-ChIP-Seq(GSE20898)/Homer(0.652) More Information \| Similar Motifs Found | motif file (matrix) |
| 36 | C G T A A C T G C G T A A C T G A C G T A C G T C G T A A C G T A G T C A C G T A C T G A C G T A G T C | 1e-21 | -5.005e+01 | 0.05% | 0.01% | 83.7bp (42.6bp) | PB0126.1\_Gata5\_2/Jaspar(0.665) More Information \| Similar Motifs Found | motif file (matrix) |
| 37 | C G A T C T A G C G T A C T G A C G A T G T C A G C T A T G A C G T A C G C T A A G T C G T A C G C A T | 1e-21 | -4.891e+01 | 0.06% | 0.01% | 89.4bp (9.7bp) | FIGLA/MA0820.1/Jaspar(0.609) More Information \| Similar Motifs Found | motif file (matrix) |
| 38 | C G T A C G T A C G T A A G T C C G T A A C T G A G T C A C G T A G T C A C T G C G T A A C T G A C T G | 1e-20 | -4.742e+01 | 0.05% | 0.00% | 80.5bp (22.5bp) | Tcf21(bHLH)/ArterySmoothMuscle-Tcf21-ChIP-Seq(GSE61369)/Homer(0.656) More Information \| Similar Motifs Found | motif file (matrix) |
| 39 | A G T C A C G T A G T C A C G T A C G T A C G T A G T C A C T G A G T C A G T C C G T A A C T G A C T G | 1e-19 | -4.403e+01 | 0.08% | 0.01% | 75.4bp (53.2bp) | PRDM1/MA0508.3/Jaspar(0.598) More Information \| Similar Motifs Found | motif file (matrix) |
| 40 | A C G T A G T C C G T A A C G T A C T G A G T C A C G T C G T A C G T A A G T C | 1e-16 | -3.745e+01 | 0.05% | 0.01% | 93.6bp (100.2bp) | Brn1(POU,Homeobox)/NPC-Brn1-ChIP-Seq(GSE35496)/Homer(0.694) More Information \| Similar Motifs Found | motif file (matrix) |
| 41 | G A T C G T C A T A C G G T A C A G C T G C T A A T G C G T A C A C T G T A G C A T C G T A G C A T G C | 1e-15 | -3.640e+01 | 0.06% | 0.01% | 86.7bp (0.0bp) | POL006.1\_BREu/Jaspar(0.646) More Information \| Similar Motifs Found | motif file (matrix) |
| 42 \* | T G A C T C G A C T G A T G C A C T A G A T G C C T A G T A C G T G A C A G T C G C A T G A T C A C G T | 1e-11 | -2.732e+01 | 0.05% | 0.01% | 78.3bp (0.0bp) | Sox9(HMG)/Limb-SOX9-ChIP-Seq(GSE73225)/Homer(0.625) More Information \| Similar Motifs Found | motif file (matrix) |
| 43 \* | A C T G A G T C A C T G A C T G A G T C A C G T C G T A A C T G C G T A A T C G | 1e-11 | -2.571e+01 | 0.06% | 0.01% | 69.5bp (3.4bp) | ZNF415(Zf)/HEK293-ZNF415.GFP-ChIP-Seq(GSE58341)/Homer(0.627) More Information \| Similar Motifs Found | motif file (matrix) |
| 44 \* | A C T G A C T G C A G T A T G C T G A C A T C G T A C G T C A G A G C T G T A C A T C G G A T C T A C G | 1e-5 | -1.161e+01 | 0.10% | 0.05% | 80.3bp (62.7bp) | HINFP(Zf)/K562-HINFP.eGFP-ChIP-Seq(Encode)/Homer(0.637) More Information \| Similar Motifs Found | motif file (matrix) |
